# Application of a fully 3D printed carbon electrode for the double potential step Chronoamperometric determination of 2,4-dinitrophenol in environmental water samples

**DOI:** 10.3389/fchem.2025.1655841

**Published:** 2025-09-03

**Authors:** Daniel Steven Shaw, Zuhayr Rymansaib, Pejman Iravani, Kevin C. Honeychurch

**Affiliations:** 1 Institute of Bio-Sensing Technology, University of the West of England, Frenchay Campus, Bristol, United Kingdom; 2 Department of High-Capacity Diagnostics, Statens Serum Institut, Copenhagen, Denmark; 3 Department of Mechanical Engineering, University of Bath, Bath, United Kingdom; 4 Centre for Biomedical Research, School of Applied Sciences, University of the West of England, Frenchay Campus, Bristol, United Kingdom

**Keywords:** 3D printed electrode, 2,4-dinitrophenol, chronoamperometry, environmental water sample, carbon nanofiber

## Abstract

**Introduction:**

The detection of nitrophenolic compounds in environmental water sources is critical due to their toxicity and persistence. This study presents the first reported application of a fully 3D printed carbon nanofiber-graphite-polystyrene working electrode for the electrochemical determination of 2,4-dinitrophenol (2,4-DNP), offering a novel and potentially cost-effective alternative to traditionally fabricated electrodes.

**Methods:**

Initial characterisation of 2,4-DNP was performed using cyclic voltammetry across a pH range of 2-8 to investigate its redox behaviour. A double potential step chronoamperometric technique was then employed, with step potentials set at −1.4 V and +0.8 V. Calibration was conducted using standard solutions of 2,4-DNP, and the method was validated using both fortified and unfortified environmental pond water samples.

**Results:**

Cyclic voltammetry revealed two reduction peaks during the initial negative scan, attributed to the reduction of the nitro groups to hydroxylamines, followed by two oxidation peaks on the positive scan corresponding to the re-oxidation of these hydroxylamines. All peaks exhibited pH dependence. The chronoamperometric calibration curve was linear over the concentration range of 50 μM to 1.0 mM (R^2^ = 0.9978), with a detection limit of 7.8 μM (S/N = 3). Analysis of pond water samples yielded a mean recovery of 106% with a coefficient of variation of 3.6% at 50 μM.

**Discussion:**

The results demonstrate that 3D printed carbon nanofiber-graphite-polystyrene electrodes are effective for the determination of 2,4-DNP in environmental water samples. The method provides reliable quantification with good sensitivity and reproducibility, highlighting the potential of additive manufacturing in the development of electrochemical sensors for environmental monitoring.

## 1 Introduction

Nitrophenols are aromatic organic compounds consisting of benzene rings, and nitro (-NO_2_) and hydroxyl (-OH) groups. The phenolic compound 2,4-dinitrophenol (2,4-DNP) has been used in the manufacture of industrial products, such as dyes, pharmaceuticals, pesticides, wood preservatives, and even in explosives with military applications. It can enter surface waters with effluents from these industries or through the use of pesticides. The accumulation of 2,4-DNP in environmental water (Dadban Shahamat et al., 2016) and air (Nojima et al., 1983) has led to the classification of 2,4-DNP as a priority pollutant by the US Environmental Protection Agency (Callahan et al., 1979). Some studies have stated its concentration in industrial effluent is around 1,000 mg/L (Dehghani et al., 2011; Paisio et al., 2009). The discharge of industrial effluents, wastewater, or pesticides contai 2,4-DNP into aquatic environments poses a significant hazard to ecosystems and public health.

The effects of nitrophenols on aquatic biota are well-documented. Exposure of fish to 2,4-DNP has been reported to negatively affect swimming performance (Marit and Weber, 2011) as well as nervous, endocrine, reproductive, and digestive systems (Kuzmina et al., 2017). The median lethal concentration (LC_50_) of 2,4-DNP varies depending on taxonomy of the organism; the LC_50_ of 2,4-DNP for freshwater algae is much higher than that of freshwater fish for example. Toxicity to plants has also been recorded in a range of studies (Shea et al., 1983) with 2,4-DNP reported to interfere with metabolic processes such as respiration and photosynthesis, and affect seed germination rates (Shaddad et al., 1989; Speer, 1973), root development (Nanda and Dhawan, 1976; Wang et al., 1967), and numerous other processes.

Studies on the absorption, distribution, metabolism, and excretion of 2,4-DNP have been performed mainly in laboratory specimens, but some information about the levels of the drug and its metabolites in humans is available from cases of occupational intoxications or ingestion of diet pills. One of the first reports of occupational intoxication with 2,4-DNP relates to the filling of armour-piercing shells with a mixture of dinitrophenol and picric acid (Perkins, 1919) during the First World War. French munition workers during the war reported problems associated with exposure to 2,4-DNP with symptoms of weight loss, weakness, dizziness, sweating, and even death by hyperthermia (Harris and Corcoran, 1995; Perkins, 1919). Subsequent investigations showed that exposure to 2,4-DNP caused these symptoms (Hargreaves et al., 2016). It does so by acting as a protonophore and dissipating the proton gradient across the mitochondrial membrane. This disrupts the proton motive force that the cell uses to synthesise the majority of its adenosine triphosphate (ATP) used to store chemical energy. The inefficiency is proportional to the dose of 2,4-DNP that is taken and results in heat generation and calorie consumption. The noted calorie consumption and subsequent weight loss led to 2,4-DNP being used as a dieting aid in the 1930s with adverse reports appearing soon afterwards (Boardman, 1935). Consequently, it was banned for human consumption in the UK, USA, and elsewhere. However, 2,4-DNP has recently been marketed as a dieting aid, principally via unregulated sales on the internet which has resulted in reports of toxicity incidents and a number of deaths (Abdelati et al., 2023; Fernardes and Izidoro, 2022; Freeman et al., 2021; Petróczi et al., 2015; Rudenko et al., 2023; Tuğcan and Kekeç, 2024). A lethal oral dose of 2,4-DNP for a human is typically considered to be 14–43 mg/kg of bodyweight (Mwesigwa et al., 2000). Nevertheless, a number of recent studies have shown that mild mitochondrial uncoupling induced by the controlled application of low-dose 2,4-DNP may have clinical applications in the treatment of neurodegenerative conditions (Geisler et al., 2017; Lee et al., 2017; Wu et al., 2017), diabetes, and steatohepatitis (Perry et al., 2015). To ensure public health security and environmental protection, there is a need for analytical techniques capable of determining 2,4-DNP in complex samples such as environmental water.

The methods used to determine aromatic nitro compounds are mainly based on gas chromatography (Xu et al., 2008) high-performance liquid chromatography (Preiss et al., 2009; Xu et al., 2022) and liquid chromatography coupled with mass spectrometry (Hofmann et al., 2008). These methods, while accurate, often have long analysis times and high running costs. Consequently, increasing attention has been paid to electrochemical methods because of their rapid operation and inexpensive instrumentation.

Aromatic nitro compounds have been shown to undergo redox reactions at various types of electrodes. These include mercury electrodes (Bermejo et al., 1993; Bratin et al., 1981; Cai et al., 1995; Lund, 1973), glassy carbon electrodes (Alemu et al., 2002; Honeychurch, 2016; Üzer et al., 2013; Xavier et al., 2021), pencil graphite electrode (Sağlam et al., 2018), carbon-fiber electrodes (Agüí et al., 2005), gold electrodes (Krausa and Schorb, 1999; Noda et al., 2001), and screen-printed carbon electrodes (Honeychurch et al., 2003; Wang et al., 1998). In previous studies, we have shown the voltammetric behaviour of 2,4-DNP at a glassy carbon electrode (Honeychurch, 2016) and its determination in serum by differential pulse voltammetry. Investigations showed that the nitro groups of 2,4-DNP can be reduced to the corresponding hydroxylamine which can be subsequently oxidised to a nitroso species. This offers a number of advantages and possibilities for the analytical determination of 2,4-DNP.

In recent years, three-dimensional (3D) printing has moved beyond its original application of industrial manufacturing and prototyping to much wider fields, including the fabrication of both physical and chemical sensors (Crapnell and Banks, 2024; Gross et al., 2017; Selemani et al., 2025). 3D printing allows for the simple fabrication of new composite designs and other important sensor components such as micro-fluidic sample handling systems (Selemani et al., 2025), analyte accumulation layers (Su et al., 2015), and even whole electrochemical cells (Gross et al., 2017). The possibility of fabricating these with high precision using robotic printing techniques has also been demonstrated (Jones et al., 2011). An additional advantage is the widespread availability of 3D printing technology through open-source platforms; allowing the advantages of creative adaption of methods and for open sharing of expertise and innovation (Kitson et al., 2016). Studies have shown the possibility of using the technology to fabricate carbon electrodes (Ambrosi and Pumera, 2016; Honeychurch et al., 2018; Pan et al., 2025; Rymansaib et al., 2016; Zheng et al., 2023), offering an attractive alternative to other carbon electrode fabrication techniques, such as screen printing and carbon paste, and an alternative to the use of expensive electrode materials, such as glassy carbon. Recently, the development of tailored filaments composed of reduced graphene oxide (rGO) and carbon black (CB) in a polylactic acid (PLA) matrix for the production of 3D printed electrochemical sensors has been reported (Silva et al., 2024). Electrodes containing rGO had improved performance (significantly lower limit of detection values) when compared with electrodes prepared in the absence of this material. The developed electrodes were applied to the detection of 2,4,6-trinitrotoluene (TNT, 96% w/w); giving a limit of detection (LoD) of 0.33 µM. A graphite/alumina/polylactic acid (G/Al_2_O_3_/PLA)-based 3D printed electrode for the electrochemical determination of TNT has also been described (Brum et al., 2025). The possibility of using square-wave voltammetry to determine TNT residues at this electrode in samples of tap water, lagoon, and seawater was explored. Recoveries of between 100.9% and 105.8% were reported. In addition, the electrode was utilised for detecting TNT residues present on different surfaces and found to allow for nanogram levels to be determined. The possibility of determining TNT in environmental samples has also recently been investigated at a 3D printed microfluidic chip (Wang et al., 2025). This incorporated a TNT biosensing complex and was reported to give a linear relationship for TNT over the range of 0.1–100 μg/mL with a limit of quantitation (LoQ) of 0.81 μg/mL.

Beyond environmental monitoring, fully 3D printed carbon-based electrodes demonstrate significant potential in biomedical sensing and industrial process control. In biomedical applications, their adaptability and low-cost fabrication enable integration into wearable and point-of-care diagnostic devices. For instance, blade-coated porous 3D carbon composite electrodes have been used in all-paper pressure sensors capable of detecting subtle physiological signals such as wrist pulses, highlighting their utility in health monitoring and rehabilitation (Zheng et al., 2024). Additionally, 3D-printed electroconductive MXene-based scaffolds have shown promise in neural tissue engineering, enhancing neurite outgrowth and neuronal differentiation under electrical stimulation (Woods et al., 2025). Electrochemical biosensors fabricated via 3D printing are also increasingly used in point-of-care testing due to their high sensitivity, portability, and affordability, enabling rapid diagnostics in decentralised settings (Bauer et al., 2021).

In industrial contexts, 3D printed electrodes offer customisable geometries and high surface areas, making them ideal for real-time monitoring of redox-active species and catalysis (Li et al., 2025). They have been applied in electrochemical processes such as hydrogen evolution (Cheng et al., 2025) and CO_2_ reduction (Padinjareveetil and Pumera, 2023), and in energy storage systems like batteries (Liu et al., 2025) and supercapacitors (Wu et al., 2025), where their porous structures enhance charge transfer and cycling stability (Kim et al., 2025). These features position 3D printed electrodes as versatile tools for advancing both clinical diagnostics and industrial process efficiency.

However, the majority of 3D printed electrodes generally require further modification and only use 3D printing to produce the base structure. Recently, it has been shown possible to fully fabricate carbon electrodes using 3D printing alone, without the need for further fabrication steps (Rymansaib et al., 2016). The determination of Pb (Rymansaib et al., 2016) and Zn (Honeychurch et al., 2018) at these electrodes fabricated from 3D printed carbon nanofiber–graphite–polystyrene has been demonstrated and we believe it is possible to determine other compounds using these same electrodes. In this present study, we investigated the cyclic voltammetric behaviour of 2,4-DNP at a fully 3D printed carbon nanofiber–graphite–polystyrene working electrode. We then explored the possibility of determining 2,4-DNP by chronoamperometry in an environmental water sample.

## 2 Materials and methods

### 2.1 Chemicals and reagents

Unless otherwise specified, all chemicals were sourced from Fisher Scientific (Loughborough, UK). Polystyrene pellets (441,147) and graphite flakes (28,286–3) were supplied by Sigma-Aldrich (Poole, UK). Acrylonitrile butadiene styrene (ABS) granules, MG94 resin were supplied by Saudi Basic Industries Corporation (SABIC; sourced from OS3DP.com). High Impact Polystyrene (HIPS) filament (HIPS175W1) was obtained from CPC Farnell (Preston, UK) and Pyrograf III carbon nanofibers from Pyrograf Products, Inc. (pyrografproducts.com, PR-24-XT HHT). Deionised water was produced using a Purite RO200-Stillplus HP System (Purite Water Purification Solutions, Oxon, UK). Stock solutions of 0.2 M orthophosphoric acid and 0.2 M trisodium phosphate were prepared by dissolving the appropriate mass in deionised water. The 0.2 M trisodium phosphate solution was titrated with the 0.2 M orthophosphoric acid solution to produce phosphate buffer solutions at pH 2, 4, 6, and 8. Stock solutions of 2,4-DNP were prepared by dissolving the required amount in acetonitrile to achieve a concentration of 10 mM. Working standards for voltammetric and chronoamperometric studies were prepared by diluting the primary stock solution with phosphate buffer and acetonitrile to achieve a final solution of 0.1 M phosphate buffer, 10% acetonitrile.

### 2.2 Apparatus

Cyclic voltammetry and chronoamperometry were undertaken using an EmStat3 potentiostat (Ivium Technologies, Eindhoven, the Netherlands) interfaced to a PC for instrument control and data acquisition. The voltammetric cell contained a carbon rod auxiliary electrode, a Ag/AgCl/KCl(3M) reference electrode, and a 3D printed carbon nanofiber–graphite–polystyrene electrode as the working electrode. The electrodes were connected to the potentiostat via suitable crocodile clips attached to coaxial cable leads inserted into the appropriate sockets on the instrument. The 3D printed carbon electrode was rinsed with deionised water, polished with tissue paper, rinsed a second time in deionised water, and dried under a stream of nitrogen gas before each scan.

### 2.3 Cyclic voltammetry

Cyclic voltammograms were recorded with solutions of 0.1 M phosphate buffer containing 10% acetonitrile, and then in the same solution containing 1.0 mM 2,4-DNP. Samples were purged with nitrogen gas (BOC, Guildford, UK) for 5 min to remove dissolved oxygen. Cyclic voltammetric investigations were undertaken using either (i) a starting and end potential of 0.0 V, an initial switching potential of −1.5 V, and a second switching potential of +1.0 V, or (ii) a starting and end potential of 0.0 V, an initial switching potential of +1.0 V, and a second switching potential of −1.5 V. The effect of the scan rate was investigated over 20 mV/s to 200 mV/s.

### 2.4 Double potential step chronoamperometry

**SCHEME 1:**
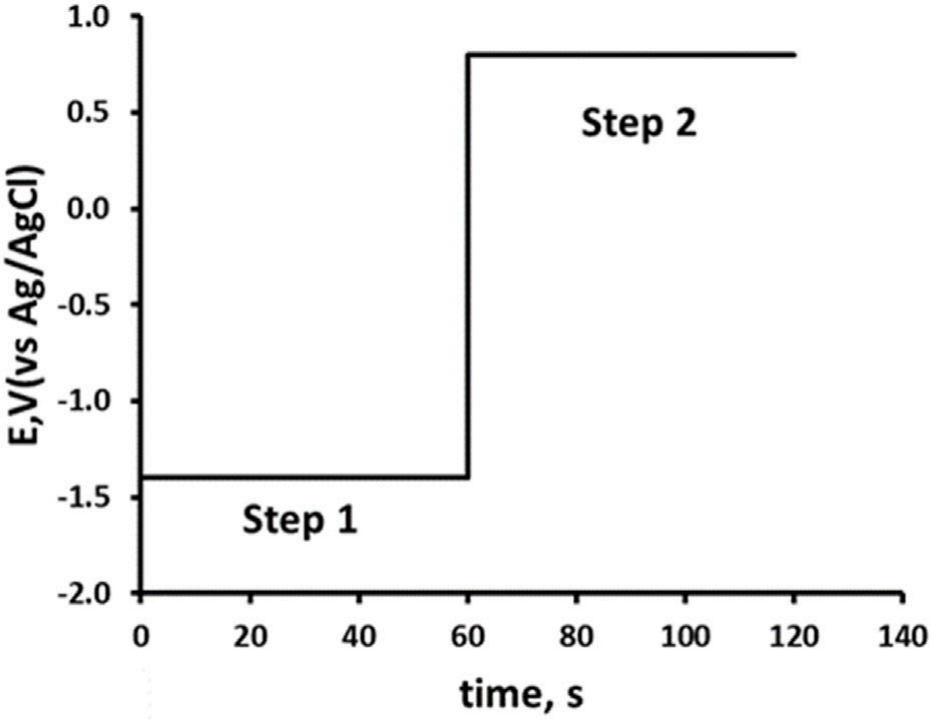
Potential waveform for double potential step amperometry. Step 1, -1.4 V from 0 to 60 s; Step 2, 0.8 V 60–120 s.

Double potential step chronoamperometric studies (Mortimer, 2017) were undertaken in solutions of 0.1 M pH 2.0 phosphate buffer, containing 10% acetonitrile using the potential waveform shown in Scheme 1. Step 1: applied potential at −1.4 V for 60 s. Step 2: applied potential of +0.8 V for 60 s. All measurements were recorded in triplicate.

### 2.5 Fabrication of electrodes

#### 2.5.1 Composite thermoplastic filament fabrication

Optimisation studies of the fabrication of the 3D-printed electrode have been published (Rymansaib, 2017; Rymansaib et al., 2016). A polystyrene/carbon nanofiber/graphite flake (80/10/10 wt%) composite was found to provide good conductivity and a stable electrochemical interface. The procedure for the preparation of the composite thermoplastic filament has also been published previously (Honeychurch et al., 2018; Rymansaib et al., 2016). To fabricate a total of 5 g of electrode material, 4 g of polystyrene (Sigma-Aldrich, Germany) were dissolved in 50 mL of chloroform (Fisher Scientific, Loughborough, UK). Separately, 0.5 g of carbon nanofibers (PR 24 XT HHT) and 0.5 g of graphite flakes (Sigma Aldrich, Germany) were sonicated in 50 mL of chloroform for 20 min. The resulting two mixtures were added to an open container and heated on a magnetic stirrer at 50 °C in a fume cupboard until the solvent fully evaporated. Once the solvent had completely evaporated, the resulting solid thermoplastic composite was placed in a heated (220 °C) 2 cm internal diameter aluminium barrel with a 2 mm internal diameter nozzle and extruded into lengths of composite conductive filament usable for 3D printing.

#### 2.5.2 Computer-aided design and 3D printing

**FIGURE 1:**
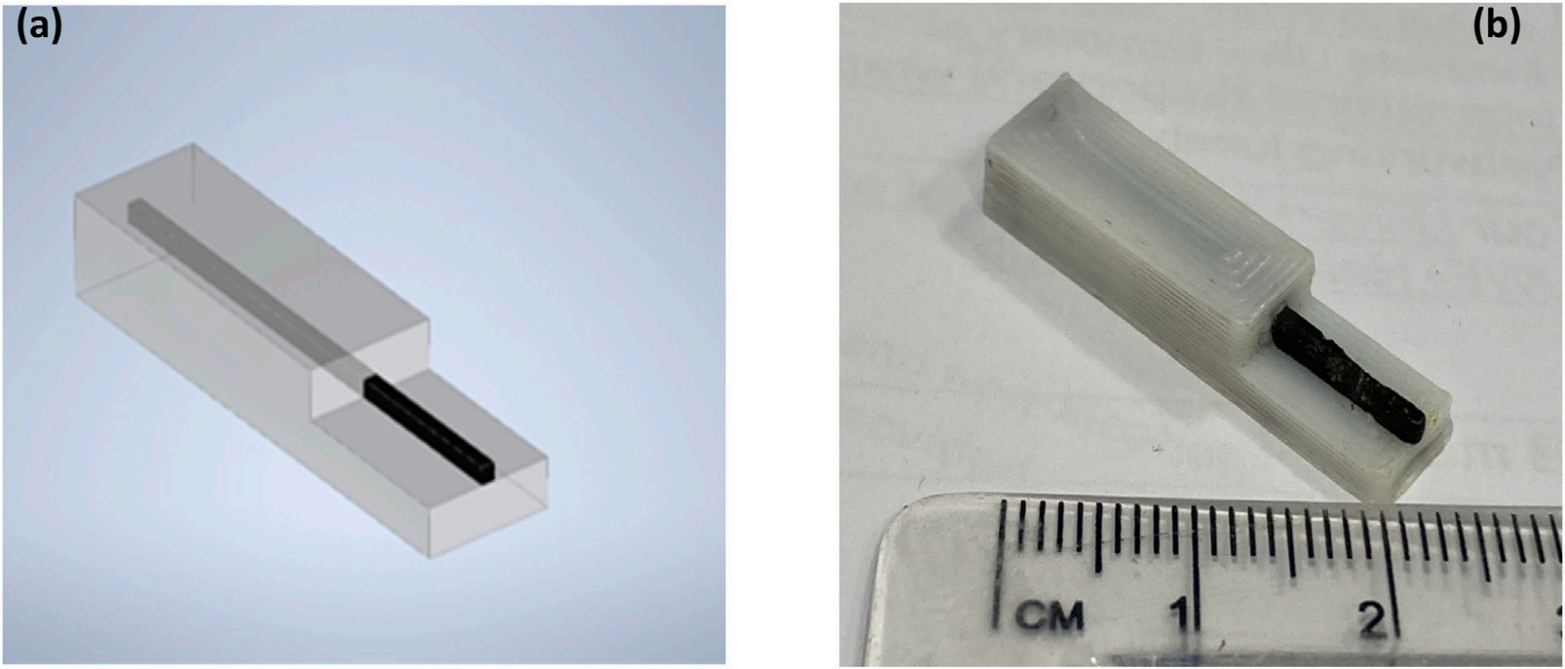
3D-printed electrode; **(a)** multi-part electrode designed in CAD software and **(b)** photograph (right) of a printed polystyrene-nanocarbon composite electrode.2.6 Analytical application.

The computer-aided design (CAD) software Solid Edge ST6 was used to design the electrode as a multi-material component. The electrode tip dimensions were 4.5 mm × 7.5 mm with a 0.656 mm^2^ active area in the centre. Printing commands were generated from the CAD design (Figure 1a) file using the open-source software Slic3r (available at slic3r.org). A layer height of 340 μm was used, resulting in nine layers from the base to the top of the conductor track for the complete electrode. The electrodes were printed using a custom-built fused filament deposition 3D printer with two 0.5 mm extruders.

Figure 1b shows a photo the fully 3D-printed carbon nanofiber–graphite–polystyrene electrode. This has been shown to exhibit superior electrochemical performance compared to conventional 3D-printed electrodes due to its synergistic material composition and tailored architecture (Rymansaib, 2017; Rymansaib et al., 2016). The integration of highly conductive carbon nanofibers and graphite creates an interconnected network that enhances electron transfer kinetics, while the polystyrene matrix ensures mechanical stability and printability without compromising conductivity. The 3D-printed structure offers increased surface area and optimised mass transport pathways, contributing to higher sensitivity and lower detection limits. Additionally, π–π interactions between the aromatic domains of the electrode and 2,4-DNP facilitate pre-concentration at the surface, further improving analytical response.

Environmental pond water samples were collected from Frenchay Campus, University of the West of England, Bristol, UK (51° 29′ 55.6″ N 2° 32′ 40.5″ W). A 4.0 mL aliquot of the environmental water sample was diluted to be 10% acetonitrile in 0.1 M phosphate buffer pH 2.0. A second aliquot of the environmental water sample was fortified to give 50 µM 2,4-DNP, and then diluted tenfold to give a 10% acetonitrile and 0.1 M phosphate buffer pH 2 solution. These were examined using the optimised double potential step chronoamperometric procedure.

## 3 Results and discussion

### 3.1 Cyclic voltammetry

**FIGURE 2:**
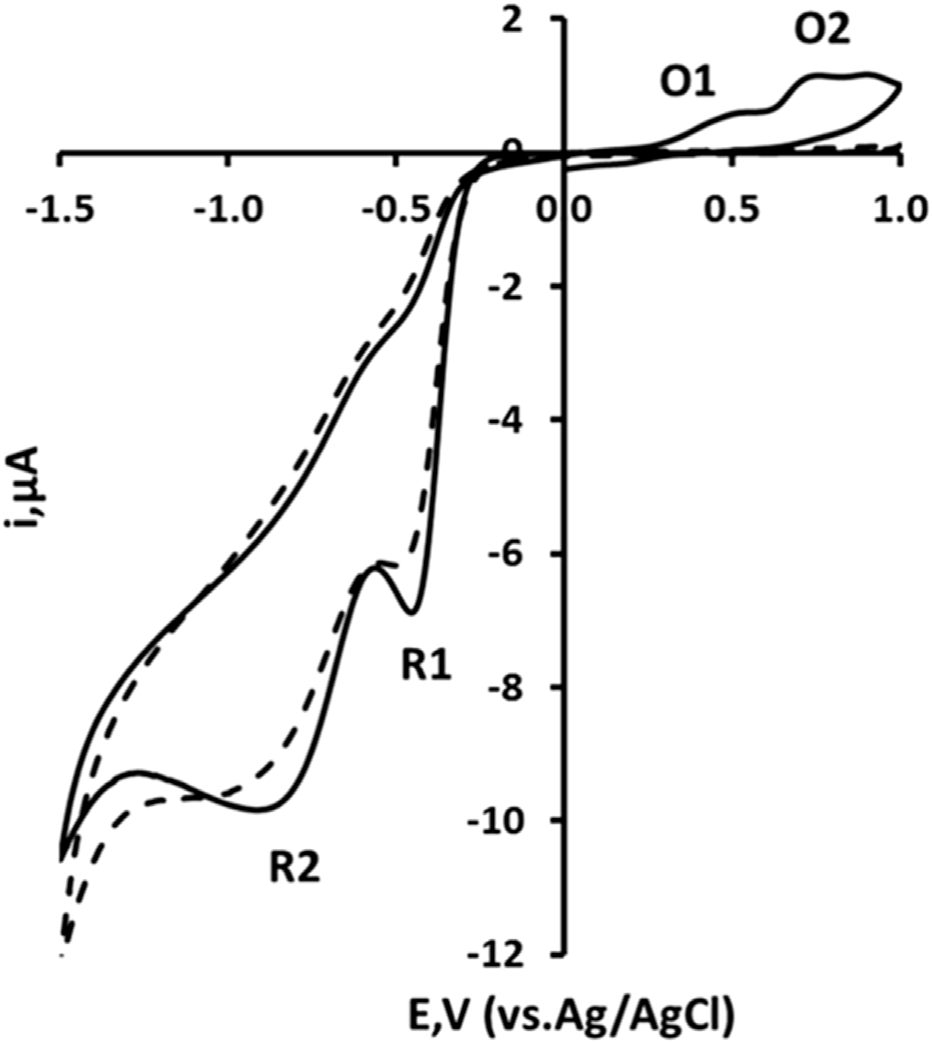
Cyclic voltammograms recorded at a 3D printed carbon nanofiber–graphite–polystyrene electrode for a 1.0 mM solution of 2,4-dinitrophenol in 10% acetonitrile, buffered with 100 mM phosphate at pH two at a scan rate of 50 mV/s for solid line: Starting and ending potential at 0.0 V, initial switching potential at −1.5 V, and second switching potential at +1.0 V. Dashed line: Starting and ending potential at 0.0 V, initial switching potential at +1.0 V, and second switching potential at −1.5 V.

The cyclic voltammetric behaviour of 2,4-DNP was studied over the range pH 2 to pH 8 (representative voltammograms obtained at pH 2 shown in Figure 2). Using an initial negative-going scan, two reduction peaks were recorded and designated R1 and R2. These are thought to result from the reduction of the two nitro groups to their corresponding hydroxylamines (Equation 1). On the subsequent positive-going scan, two oxidation peaks are observable (designated O1 and O2) corresponding to the oxidation of the electrochemically-generated hydroxylamine to the corresponding nitrosamine (Equation 2).

$$R-{\text{NO}}_{2}+4e^{-}+4H^{+}\;\to \;R-N\left(H\right)-\text{OH}+H_{2}O$$ (1)

$$R-N\left(H\right)-\text{OH }\to \;R-N=O+2e^{-}+2H^{+}$$ (2)

Further studies were undertaken to investigate this proposed mechanism. If the voltammetric scan was first implemented in a positive direction, no oxidation peaks were observable. Thus, the oxidative responses did not occur unless the molecule had undergone some previous reduction process. Hence, it was concluded that the two oxidation peaks result from the oxidation of the hydroxylamine (Equation 2) formed via the process described in Equation 1.

### 3.2 Effect of pH on peak potential

**FIGURE 3:**
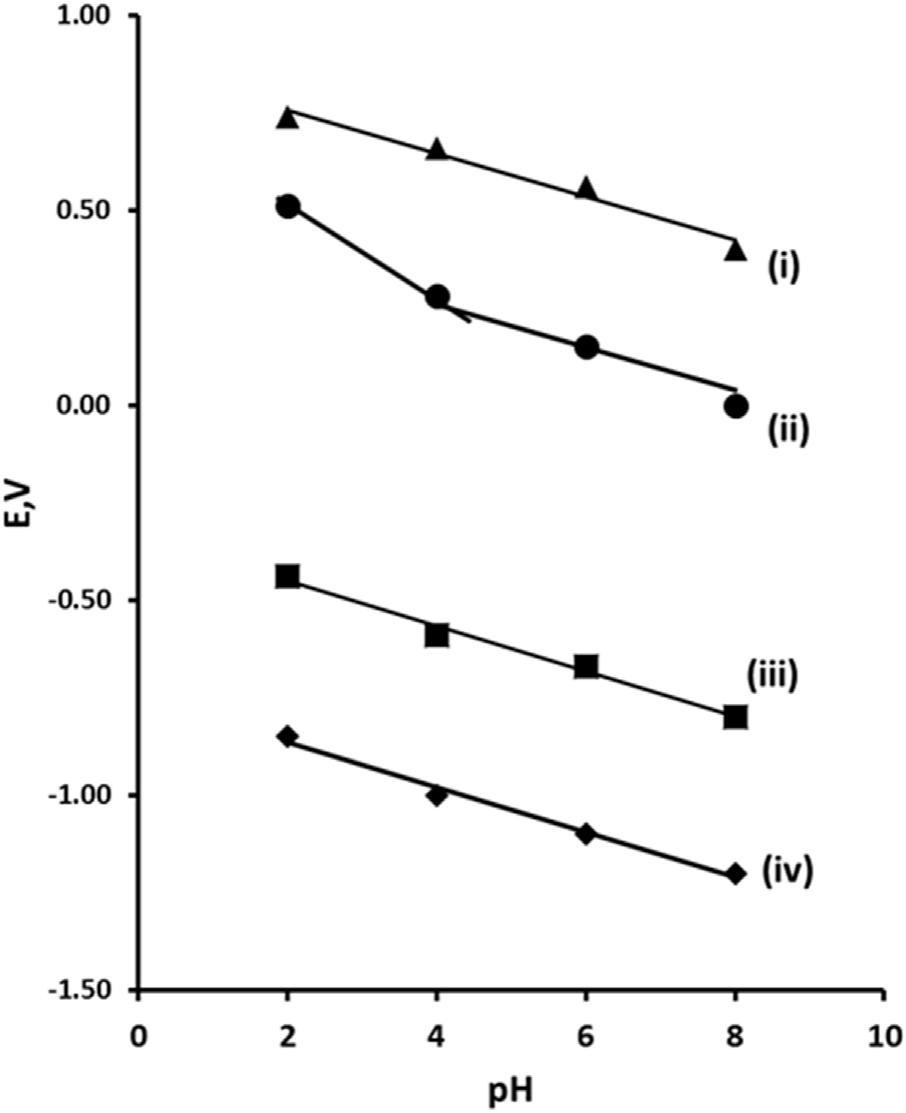
Plot of Ep vs pH for 2,4-dinitrophenol. (i) peak O2, (ii) peak O1, (iii) peak R1, (iv) peak R2. Error bars omitted as the reproducibility of Ep across replicates resulting in standard deviations that would not be visually prominent on the plot at the presented scale.

The peak potential (Ep) for peaks R1, R2, O1, and O2 was obtained over the range pH 2–8 (Figure 3). All of the peaks observed were found to be pH dependent. Plots of the Ep values of peaks R1, R2, and O2 against pH were found to give slope close the Nernst theoretical values of 59 mV/pH indicating an equal number of protons and electrons were involved in their reductions or oxidations. However, peak O1 was found to have a break point at pH 4.1. It is possible that this is a result of acid dissociation (pKa) associated with the phenol group. The pKa of 2,4-dinitrophenol is 4.13 (Pearce and Simkins, 1968) which is close to the result obtained. Consequently, we believe that oxidation process seen at O1 is related to the oxidation of the phenolic group. However, this oxidation is not observable without prior reduction of the molecule. This was concluded to be due to the stabilisation of the phenolate anion by the electron withdrawing properties of the hydroxylamine groups formed from at higher pH values. At pH values below the pKa, this stabilising effect is removed allowing for the oxidation of phenol. This effect can be seen in the change in the slope of the plot of Ep verses pH for O1 shown in Figure 3. At pH values above pH 4 a slope of 55 mV/pH unit is results, close to the theoretical Nernst value for an oxidation process involving an equal number of electron and protons. However, a slope of 120 mV/pH unit is recorded below pH 4; indicative of an oxidation process involving a two to one ratio of protons to electrons. We believe that this is the result of the loss of the stabilising effect of the electron withdrawing nitro groups in the prior reduction step. At this lower pH and without stabilisation the nitro groups are reduced to their corresponding amine groups. As amines act as electron donating groups, their presence allows for the oxidation of the phenol to a radical, and then via the loss of a further proton from one of the amine groups to a residence stabilised phenolate. The possibility of direct electrochemical oxidation of 2,4-DNP via one-electron oxidation of the phenolic group has been demonstrated previously (Yin et al., 2012). However, we have not seen evidence for this in the cyclic voltammetric investigation conducted here. Both of the reduction processes (R1 and R2) showed slopes of 55 mV/pH unit; this is close to the theoretical Nernst value for reduction processed involving an equal number of electron and protons.

### 3.3 Double potential step chronoamperometry

Chronoamperometry offers the possibility of simple operation and utility outside of the laboratory with small sample volumes. In comparison to other voltammetric techniques, chronoamperometry can give an improved signal-to-noise ratio as the current can be integrated over longer time intervals. As we were interested in determining low concentrations of 2,4-DNP, we chose to investigate the possibility of utilising a chronoamperometric measure step for the determination of 2,4-DNP. We focused our investigations on the anodic oxidation of the electrochemically generated hydroxylamine species (Equation 2). This offers analytical advantages, as it avoids interference from the reduction of sample components, such as oxygen. The double step amperometric measurement (Mortimer, 2017) step is potentially open to interferences from the redox behaviour of metal ions, such as Pb^2+^ and Cu^2+^. However, as previously shown (Honeychurch, 2019), metal ions exhibit poor electrochemical activity in phosphate-based electrolytes like those employed here. Primarily Na^+^ and K^+^ salts are the only ones readily soluble in water and are not electrochemically active under the conditions employed here. The anodic region, particularly at low positive potentials, offers several analytical advantages for electrochemical detection. Background currents in this region are typically lower than those observed in the cathodic range, resulting in an improved signal-to-noise ratio and enhanced sensitivity. Additionally, fewer electroactive species are present at these potentials which reduces the likelihood of signal overlap and interference thereby improving the clarity and reliability of the analytical response. Oxidation processes, especially those involving pre-generated intermediates such as hydroxylamines, also tend to produce more stable and reproducible signals compared to direct reductions in complex environmental matrices.

**FIGURE 4:**
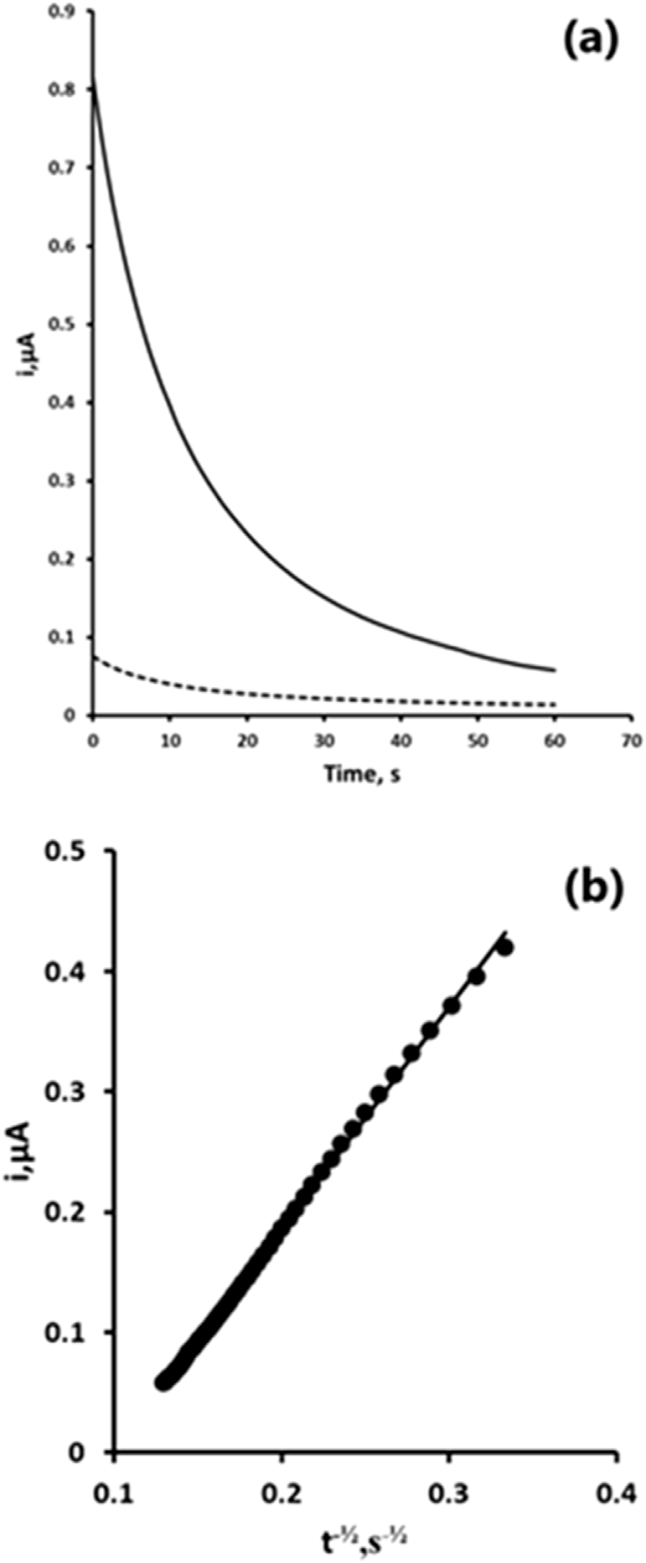
**(a)** Chronoamperometric response obtained in the presence (solid line) and absence (dashed line) of 1 mM 2,4-DNP; **(b)** Cottrell plot of 1 mM 2,4-DNP obtained from the chronoamperometric response **(a)**.

We first investigated the chronoamperometric behaviour of a quiescent 10% acetonitrile 0.1 M pH two phosphate buffer in the presence and absence of 1 mM 2,4-DNP. A potential of −1.4 V was applied for 30 s to reduce 2,4-DNP to its corresponding hydroxylamine (Equation 1). The potential was step to +1.0 V and held for 60 s to oxidise the hydroxylamine formed to the nitrosamine, giving the analytical signal (Figure 4a). Plots of current (i) vs the inverse of the square root of time (t^−½^) showed the response to follow Cottrell type behaviour (Figure 4b) (Pletcher et al., 2001).

### 3.4 The effect of pH on the chronoamperometric response

**FIGURE 5:**
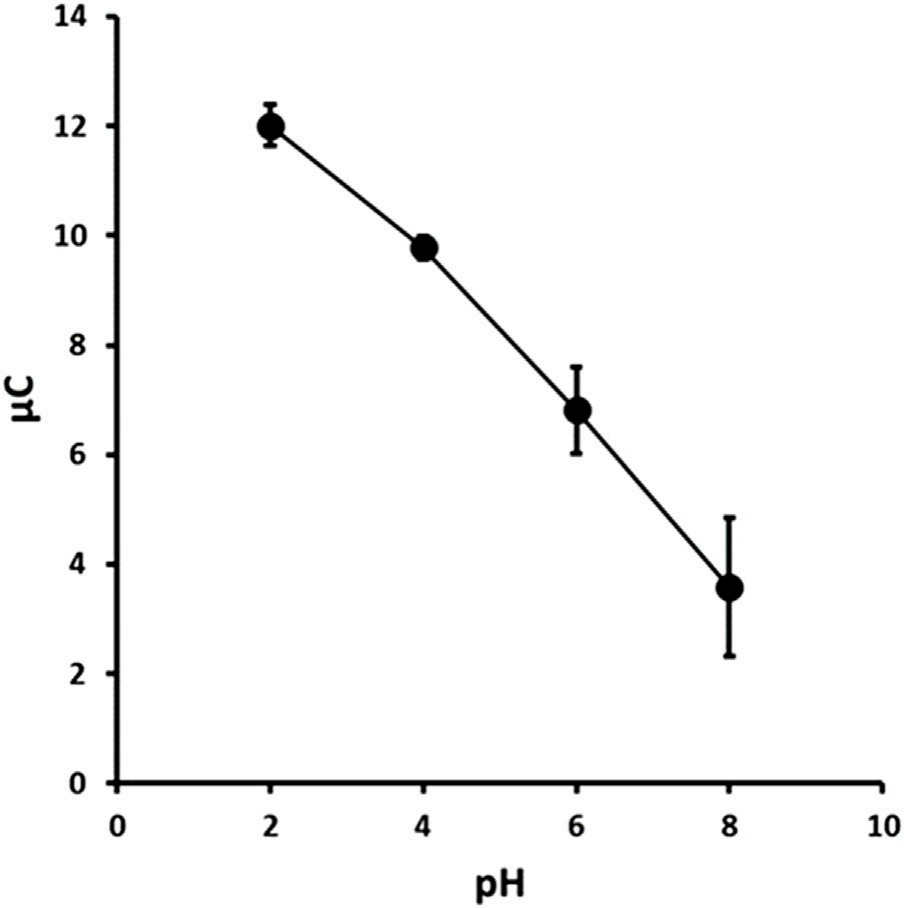
Chronoamperometric response versus pH for 1.0 mM 2,4-DNP. Each point is the mean of three separate measurements. Error bars represent ± σ.

The chronoamperometric current response was found to be pH dependent over the range studied (Figure 5) and was found to decrease in a near linear decrease (R^2^ = 0.994, −1.395 μC/pH) with increasing pH. This was likely due to the stabilisation of the nitro groups in 2,4-DNP through charge delocalisation facilitated by the phenolate anion. The maximum peak current of the oxidation peak O1 was obtained at pH 2.0. Therefore, subsequent studies were undertaken using a supporting electrolyte of 0.1 M pH 2.0 phosphate buffer.

### 3.5 Optimisation of chronoamperometric step potentials

**FIGURE 6:**
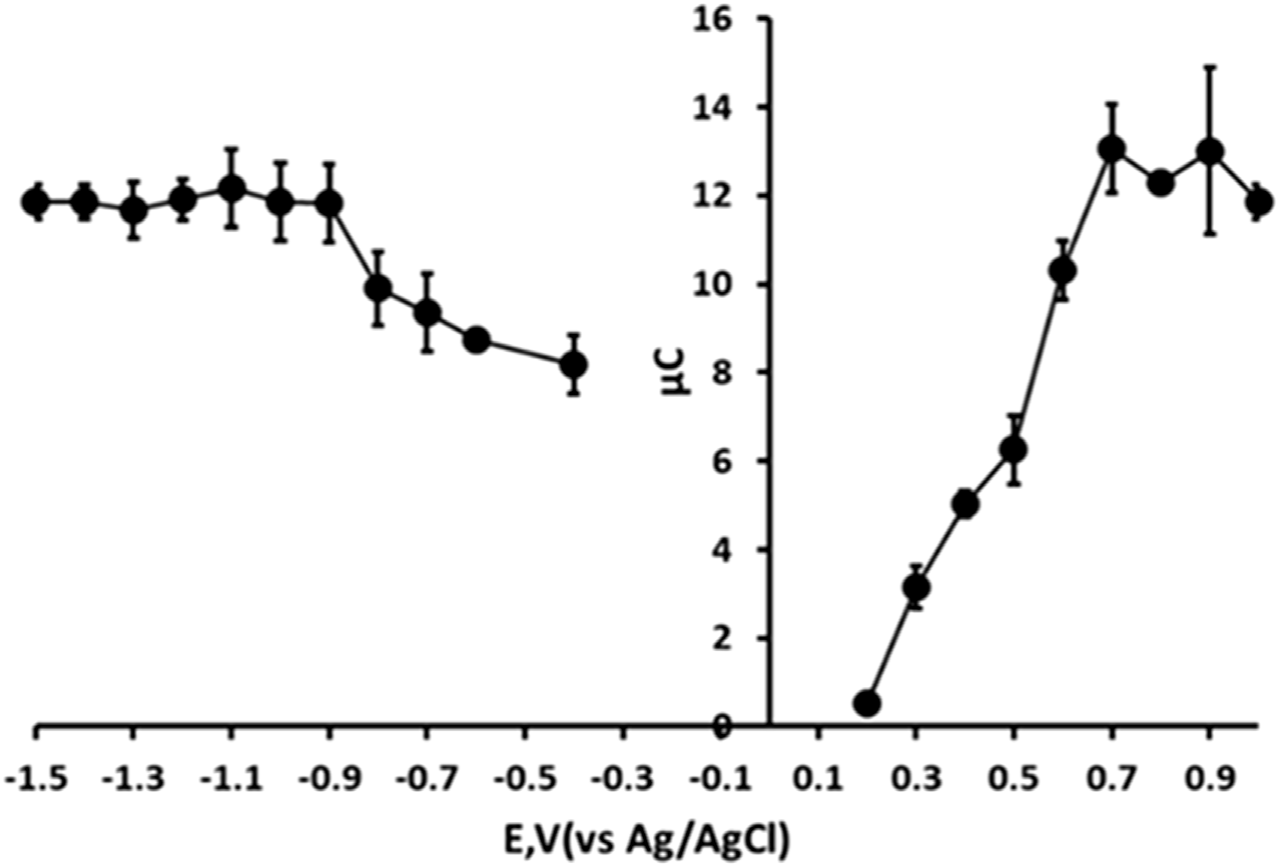
Effect of reduction and oxidation step potentials. Each point is the mean of three separate measurements. Error bars represent ± σ.

We first optimised the applied reduction potential used in the first step of the chronoamperometric measurement. The effect of this reduction potential was investigated over the range −0.4 V to −1.5 V by monitoring the resulting oxidation current recorded at +1.0 V (Figure 6). The oxidative chronoamperometric response was found to increase with increasing negative potential over the range −0.4 V to −0.9 V. At more negative potentials the oxidative response was found to reach a constant. However, as the potential beyond −1.0 V was made more negative the precision of the measurement improved with the lowest coefficient of variation (3.2%) being obtained at −1.4 V. Consequently, an applied potential of −1.4 V was used for step 1 (reduction) of the chronoamperometric measurement in further studies.

Subsequently, the oxidative measurement step (step 2) was examined. The effect of applied potential was studied over the range +0.2 V to +1.0 V. In this investigation, the resulting chronoamperometric signal was found to increase with increasing positive potential over the range +0.2 V to +0.8 V. The signal was found to become constant at more positive potentials and so further studies were undertaken using an applied step 2 potential of +0.8 V.

### 3.6 The effect of time on the chronoamperometric response

**FIGURE 7:**
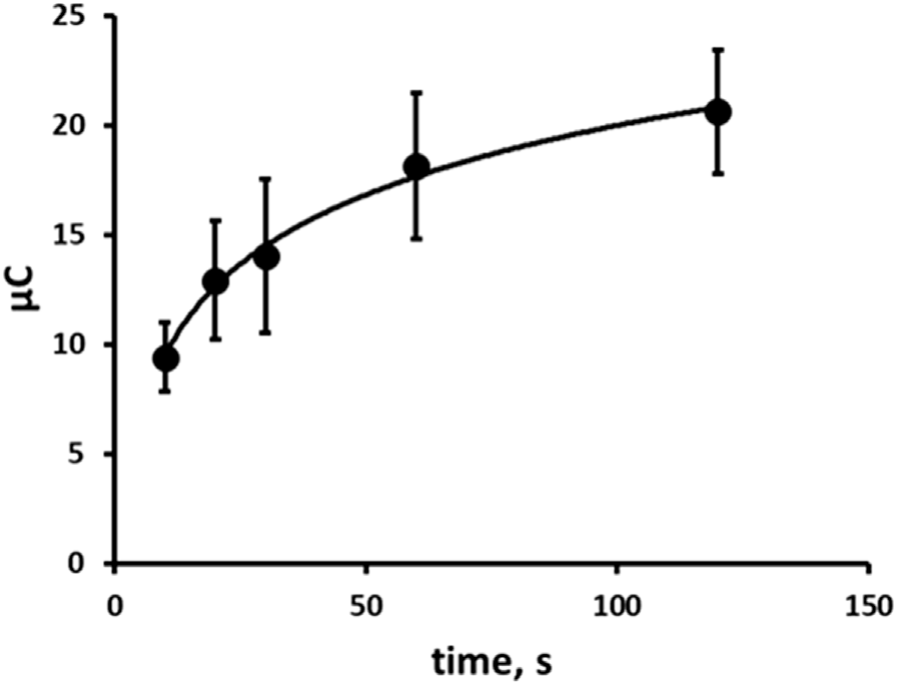
Effect of reduction step time on resulting chronoamperogram. Each point is the mean of three separate measurements. Error bars represent ± σ.

The effect of step 1 reduction time in an unstirred, quiescent solution on the resulting oxidative chronoamperometric response was investigated over the range 10 s–120 s. The oxidation signal increased with time and exhibited a logarithmic relationship (Figure 7). In this proof-of-concept investigation based on a balance of time against sensitivity, a step 1 reduction time of 60 s was selected and used in further investigations.

### 3.7 Calibration curve and limit of detection

Initial studies were undertaken to study the effect of 2,4-DNP concentrations on the magnitude of the chronoamperometric response. The calibration plot was found to be linear from 50 μM to 1.0 mM (0.4399 μA/mM; R^2^ = 0.9980), with a detection limit of 7.8 µM (based on a signal-to-noise ratio of three). A coefficient of variation of 4.7% was obtained for a 0.05 mM 2,4-DNP solution. The LOD was calculated based on measurement of the blank signal; the signal measured when no analyte is present. This was determined using the following equation (Equation 3):

$$LOD=\frac{3\;\sigma_{blank}\;}{S}$$ (3)

### 3.8 Analytical application

**FIGURE 8:**
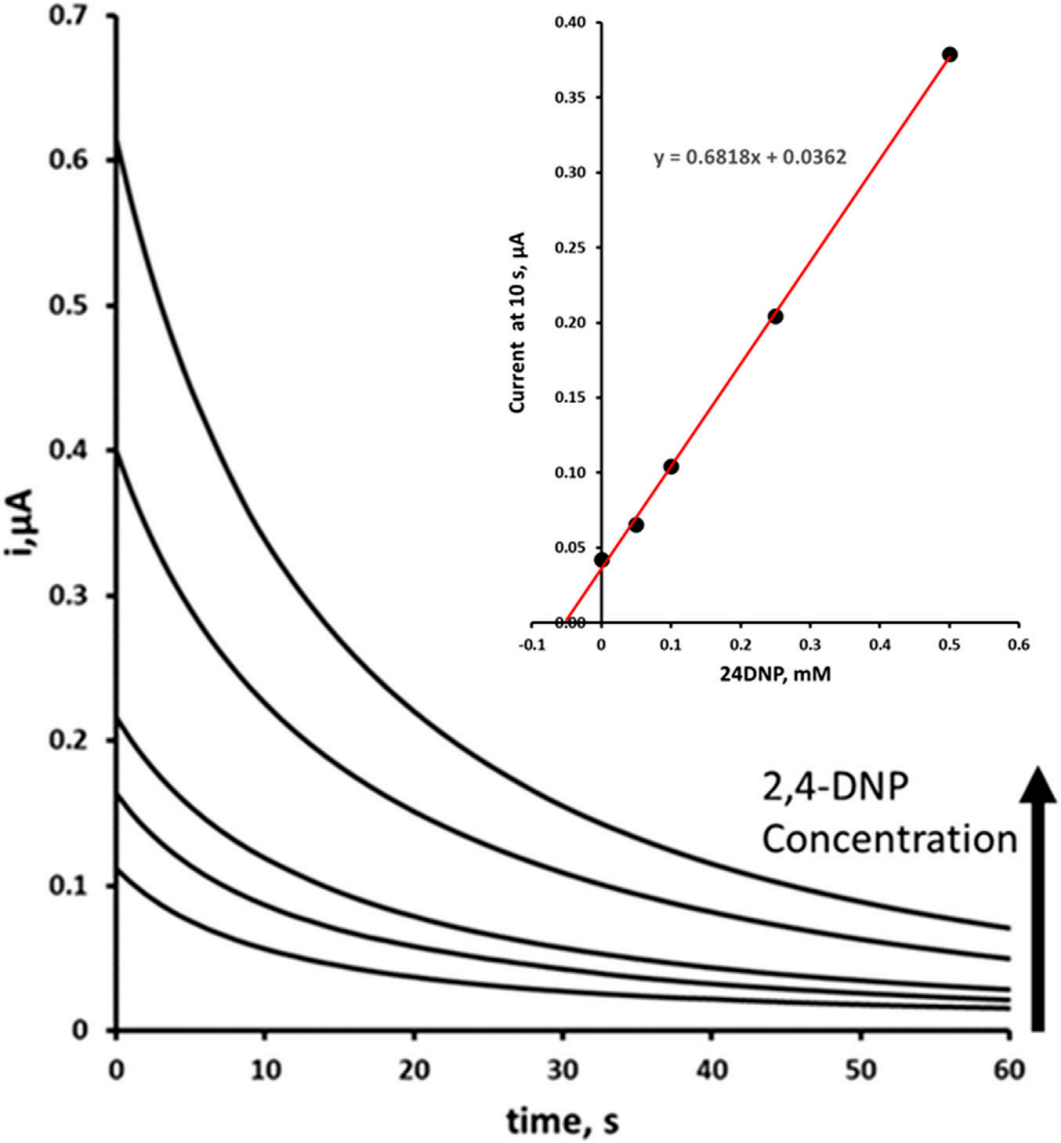
Double step amperometric determination of 2,4-DNP by multiple standard addition in environmental pond water sample fortified at 0.05 mM 24DNP plus additions of 0.0, 0.05, 0.1, 0.25, and 0.5 mM. The analysis was performed using a two-step redox process with a Step 1 reduction potential of −1.4 V and a Step 2 oxidation potential of +0.8 V. Insert blank corrected multiple standard addition plot.

The optimised procedure was used to determine the concentration of 2,4-DNP in fortified and unfortified environmental pond water samples (typical amperograms shown in Figure 8). A mean recovery of 106% was obtained for a 0.05 mM 2,4-DNP fortified environmental pond water sample with an associated coefficient of variation of 3.6%. These results show that the optimised chronoamperometric method is capable of determining 2,4-DNP in environmental water samples.

The findings demonstrate that these 3D-printed working electrodes serve as effective alternatives to those made from conventional materials, providing reliable data for the determination of 2,4-DNP. The performance characteristics are similar to those previously reported at traditional electrode materials such as Hg modified Ag electrodes with detection limits ranging between 2.7 µM (Fischer et al., 2007) to 10 µM (Danhel et al., 2009) being reported. Notably, the detection limit is an improvement on that at Pt and Au electrodes of 85 µM (Khachatryan et al., 2005). A number of reports have shown that lower limits of detection can be obtained at carbon-based electrodes fabricated from more traditional materials such as glassy carbon. However, these have utilised secondary modifications to the electrode surface; including surfactants (Wang et al., 2006), graphene and molecularly printed polymers (Liu et al., 2012), metal nanoparticles (Yang et al., 2011), and sample preparation steps such as solid phase extraction (Lezi et al., 2014) and liquid chromatography (Karaová et al., 2016; Ruana et al., 1993). A dual-function 3D printed electrochemical device based on recycled PLA combined with carbon black, and graphite that acts both as a collector and detector has recently been developed (Souza et al., 2024). Using square-wave voltammetry for the determination of TNT, limit of detections of between 0.88 and 3.42 µM were reported. They demonstrated the possibility of determining TNT residues on a metal surface following an explosion by either rubbing the electrode over the surface or by tapping the electrode on the contaminated surface. Similarly, a 3D printed electrochemical sensor made from a graphite/polylactic acid (Gpt-PLA) composite was evaluated using square-wave voltammetry for the detection of TNT in environmental water samples (Siqueira et al., 2023). Three reduction peaks with two being selected for quantification giving limits of detection of 0.52 μM and 0.66 μM. Using reduction processes as the analytical signal can be problematic as commonly occurring sample components, such as oxygen and metal ions, can interfere. Our reported approach could offer some advantages over this.

## 4 Conclusion

This is the first example of the voltammetric behaviour and chronoamperometric determination of 2,4-DNP at a fully 3D printed carbon nanofiber–graphite–polystyrene electrode. A simple double potential step chronoamperometric assay for 2,4-DNP was developed and well-defined amperometric signals can be obtained with a detection limit of 7.8 µM and a linear response from 50 µM up to 1.0 mM (R^2^ = 0.9978). A mean recovery of 106% (% CV = 3.6%) was obtained for an environmental water sample fortified with 50 µM 2,4-DNP. An important consideration in the development of the chronoamperometric method was the potential for interference from common electroactive species present in environmental water samples. By focusing on the anodic oxidation of the electrochemically generated hydroxylamine species, the method inherently avoids interference from species such as dissolved oxygen and reducible metal ions, which typically affect cathodic measurements. This strategic choice enhances the selectivity of the method for 2,4-DNP in complex matrices. Future work could explore the adaptation of this method for multi-analyte detection using modified electrodes and chemometric analysis, enabling broader applicability in environmental monitoring.

## Data Availability

The raw data supporting the conclusions of this article will be made available by the authors, without undue reservation.
